# Comparison of recurrence patterns in cervical cancer patients with positive lymph nodes versus negative

**DOI:** 10.1002/cam4.4860

**Published:** 2022-05-26

**Authors:** Mei Ji, Yuan Liu, Yan Hu, Jing Sun, Haiyan Zhu

**Affiliations:** ^1^ Department of Gynecology, Shanghai First Maternity and Infant Hospital, School of Medicine Tongji University Shanghai China; ^2^ Department of Gynecology The First Affiliated Hospital of Wenzhou Medical University Zhejiang China

**Keywords:** cervical cancer, lymph node status, patterns of recurrence, risk of recurrence

## Abstract

**Purpose:**

The aim of this study was to compare patterns of recurrence in 2009 FIGO Stage IB‐IIA (T_1b_N_0_M_0_‐T_2a_N_0_M_0_) cervical cancer patients with cN0 and cN1.

**Methods:**

The epidemiological and clinical data of 1352 patients who had undergone radical hysterectomy and systematic lymphadenectomy with cervical cancer treated from January 2008 to April 2019 at a tertiary teaching hospital were retrospectively collected. The primary aim was to discover the lymph node status‐dependent patterns and time of recurrence.

**Results:**

Tumor recurrence and death were significantly less common in patients with cN0 than cN1. In addition, the length of time to recurrence (median 60 months cN0 vs. 43 months cN1, *p* < 0.001) and death (median 84 months cN0 vs. 68 months cN1 *p* < 0.001) were significantly longer in cN0 versus cN1 patients. The cumulative rate of relapse also showed a significant difference between cN0 and cN1 groups, especially the 1‐year relapse rate (2.14% vs. 10.78%). Of the patients who recurred, there was no difference in number of recurrent sites between cN0 and cN1 groups (solitary metastases:35.8% of cN0 and 35.6% of cN1; multiple metastases: 64.2% of cN0 and 64.4% of cN1). Similarly, there was no statistical difference in recurrence sites of cervical cancer between cN0 and cN1 groups based on three categories, (*p* = 0.646). However, in the six categories, patients' vaginal vaults with negative lymph nodes were more prone to recurrence, while the distribution of other recurrence sites showed no significant difference between the two groups.

**Conclusions:**

There is a significant improvement of relapse‐free survival in the cN0 group, and the recurrence time of cN0 patients is significantly delayed than cN1 group. However, except for the risk of metastasis of the vaginal vault, the site of relapse remains similar.

## Graphical abstract

This is the first report to explore the recurrence patterns in cervical cancer patients with positive lymph nodes versus negative. Interestingly, sites of recurrence were similar, except for the risk of vaginal vault metastasis. However, the time to recurrence and overall survival showed significantly longer in the cN0 group compared to the cN1 group. Our research was intended to provide clinical evidence on the necessity and feasibility of accurate follow‐up according to lymph node status.

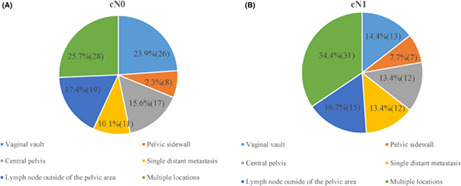

## 1 INTRODUCTION

Cervical cancer is one of the most common cancer diagnosed among women and the fifth leading cause of cancer death among women worldwide.^1^ In 2015, approximately 311,365 women were diagnosed with cervical cancer and the disease resulted in 30,500 deaths in China.^2^ The recurrence rates of International Federation of Gynecology and Obstetrics (FIGO) Stage IB–IIA and IIB–IVA cervical cancer are 11% to 22% and 28% to 64% respectively, with a standard treatment strategy.^3^ Lymph node metastasis is one of the most important factors for relapse and poor clinical outcomes.^4^, ^5^, ^6^, ^7^, ^8^

In FIGO Stages IB–IIA, the 5‐year survival rates with lymph node metastasis and without lymph node metastasis are 51%–78% and 88%–95%, respectively.^9^, ^10^ The recurrence rate of lymph node‐positive patients is 27%, while that of lymph node‐negative patients is 10%.^11^ In conclusion, patients with positive lymph nodes (cN1) have a significantly higher risk of recurrence than patients with negative lymph nodes (cN0); however, it is unclear whether the pattern of recurrence, including the timing and site of recurrence, is the same for cN1 and cN0 groups. It is well known that the main purpose of tumor follow‐up is to detect patients with recurrence and metastasis at an early stage. Based on the concept of precision medicine, should the interval and items of follow‐up be different between the cN1 and cN0 groups? In this large population‐based analysis with long‐term follow‐up, we aim to investigate recurrence sites and time patterns in cervical cancer patients with positive lymph nodes and negative, in order to explore individualized follow‐up protocols based on lymph nodes status.

## 2 METHODS

### 2.1 Study cohort

This study was approved by the Ethics Committee of the First Affiliated Hospital of Wenzhou Medical University and informed consent was signed by the patients before surgery. Data were collected from a retrospective database based on the cervical cancer patients undergoing radical hysterectomy and pelvic lymphadenectomy at the First Affiliated Hospital of Wenzhou Medical University between January 2008 and April 2019. Cervical tissues were histologically confirmed by two pathologists. The tumor stages were assessed according to the International Federation of Gynecology and Obstetrics (FIGO) staging system.^12^ The histological grades were classified according to the World Health Organization criteria. Patients with either a minimum of 6 months follow‐up or evidence of recurrence were included in the study. Patients with metastatic cervical cancer, those who died 6 months after surgery, and those with stage IA1 (T_1a1_N_0_M_0_) without intraoperative lymph node dissection were excluded. Clinicopathologic variables collected include age at operation, surgical routes, histological type, 2009 FIGO stage, histological grade, adjuvant therapy, depth of stromal infiltration, tumor size, lymphovascular space invasion (LVSI), positive vaginal margin, and parametrial extension. Postoperative outcomes include the date of first recurrence, site of the first recurrence, and overall survival. Follow‐up visits were conducted every 3 months for the first 2 years, every 6 months for the following 3 years, and once a year thereafter.

The primary outcome of the research was to identify the relapsing patterns of different lymph node status of cervical cancer by assessing the time and site of recurrence. Recurrent disease was assessed by physical examination, imaging techniques, and biopsy when feasible. Disease‐free survival (DFS) was defined as the interval from the date of surgery to proven local recurrence or distant metastasis. According to previous reports,^13^, ^14^ the patients were classified into six and three categories by the site of relapse as follows: six categories were specifically divided into vaginal vault, pelvic sidewall, central pelvis, single distant metastasis, lymph node outside of the pelvic area, and multiple locations. Three categories were specifically divided into within pelvic cavity, beyond pelvic cavity, both within and beyond pelvic cavity. The secondary outcome was to identify the lymph node status‐dependent DFS and overall survival (OS). The follow‐up ended with the patient's death, the last information available in the tumor registry, or their last follow‐up appointment before the study closing date (October 31, 2020).

### 2.2 Statistical analysis

Statistical analysis was performed using SPSS Version 26. The patient's characteristics and clinicopathological factors were analyzed using Chi‐square statistics. DFS and OS were calculated as the median of actual survival from the time of diagnosis, and log‐rank test was used to compare the differences among the different groups in terms of DFS and OS. Survival analysis was based on the Kaplan–Meier method. Multivariable analysis was performed with the Cox proportional hazard model to evaluate independent factors possibly affecting survival. Criteria for inclusion were significant on univariate analysis, and *p* < 0.05 was considered significant.

## 3 RESULTS

### 3.1 Characteristics between cN0 and cN1

**FIGURE 1:**
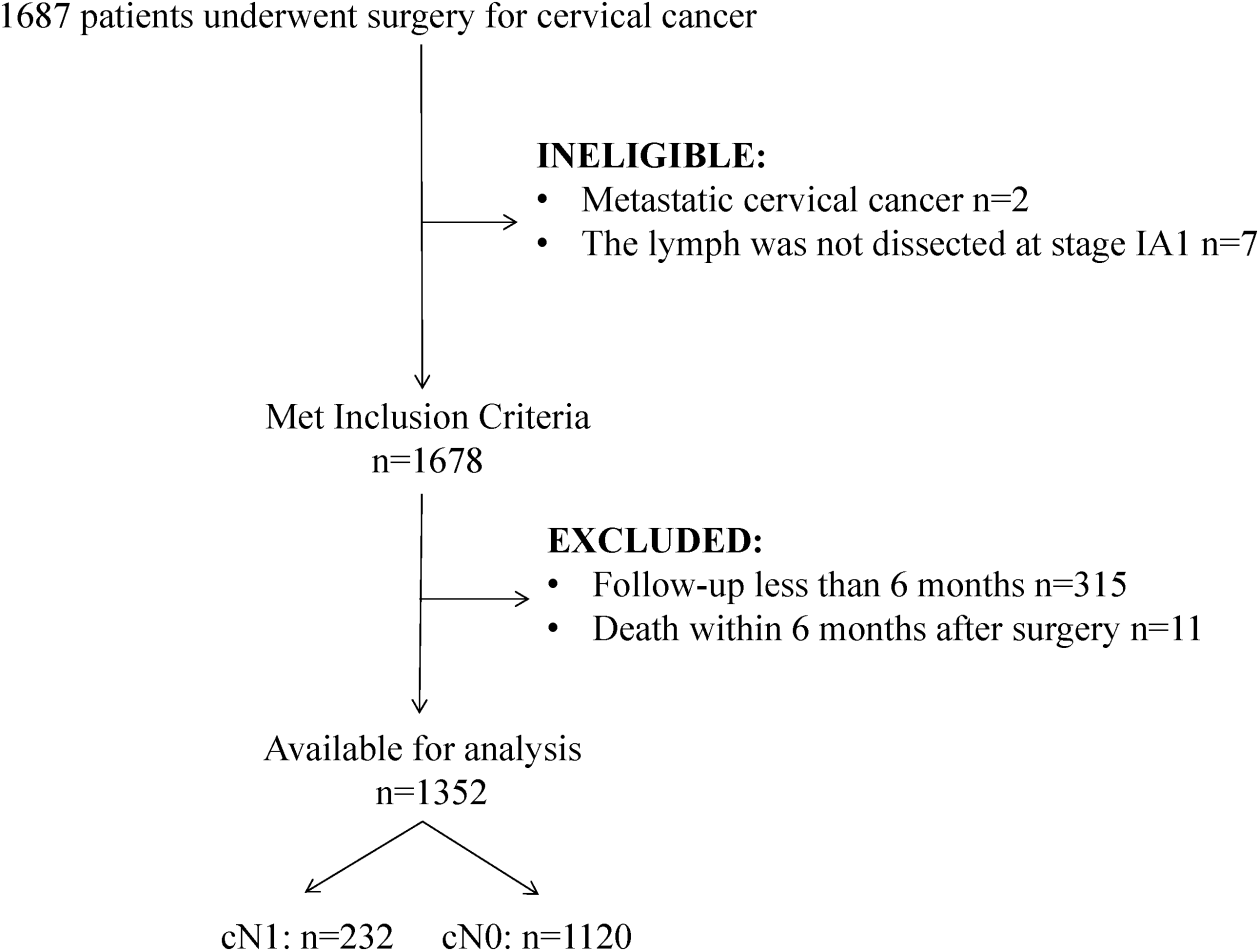
Flowchart of study population

A total of 1352 patients with cervical cancer met the inclusion criteria (Figure 1). Clinicopathologic characteristics of patients are described in Table 1. Among them, 1120 (82.8.%) showed negative lymph nodes (cN0) and 232(17.2%)showed positive lymph nodes (cN1). The most common clinical stage according to the 2009 FIGO staging system was Stage I(T_1_N_0_M_0_) (59.8%). However, Stage II(T_2_N_0_M_0_) cases were significantly more common in cN1 group than in cN0 group (58.6% vs. 36.3%, *p* < 0.001). In both groups, the most common histologic type was squamous cell carcinoma (SCC). In our research, the majority (47.8%) of tumors were poorly differentiated, whereas cN0 patients displayed more well‐differentiated tumor than cN1 patients (*p* < 0.001). Patients with cN1 disease were more likely to have larger cancers (tumor size ≥ 4 cm: 43.1% vs. 12.2%, *p* < 0.001). CN1 patients had deeper stromal infiltration (stromal infiltration≥1/2: 90.1% vs. 48.9%, *p* < 0.001) and were more likely to have a positive resection margin than patients with cN0 disease (4.7% vs. 2.2%, *p* < 0.001). Apart from that, lymphovascular invasion and parametrial extension were also more frequent in cN1 cancers (cN1 49.6% vs. cN0 16%, *p* < 0.001 and cN1 10.8% vs. cN0 0.5%, respectively *p* < 0.001). In both cohorts, the majority of patients received adjuvant treatment (62.1%), cN1 patients were more likely to receive adjuvant therapy than cN0 patients (71.6% vs. 60.2%, *p* = 0.001). Chemotherapy alone and chemoradiation were the most common types of adjuvant therapy (19.1% and 30.5% respectively), while radiation alone was less common, accounting just for 12.5%.

**TABLE 1:** Relationships of lymph node status with clinicopathological factors of cervical cancer

Variables	Median/*n*	IQR(%)	Median/*n*	IQR(%)	Median/*n*	IQR(%)	*p‐*value
Age (years)							0.078
<45	313	23.2%	249	22.2%	64	27.6%	
≥45	1039	76.8%	871	77.8%	168	72.4%	
Surgical routes							0.001
Laparoscopy	103	7.6%	98	8.8%	5	2.2%	
Open surgery	1249	92.4%	1022	91.2%	227	97.8%	
Histological type							0.538
SCC	1165	86.2%	959	85.6%	206	88.8%	
Adenocarcinoma	128	9.5%	111	9.9%	17	7.3%	
Adenosquamous carcinoma	40	2.9%	33	3.0%	7	3.0%	
Special	19	1.4%	17	1.5%	2	0.9%	
FIGO stage							<0.001
I	809	59.8%	713	63.7%	96	41.4%	
II	543	40.2%	407	36.3%	136	58.6%	
Histological grade							<0.001
Well differentiated	202	14.9%	192	17.2%	10	4.3%	
Moderately differentiated	504	37.3%	416	37.1%	88	37.9%	
Poorly differentiated	646	47.8%	512	45.7%	134	57.8%	
Adjuvant therapy							0.001
No	512	37.9%	446	39.8%	66	28.4%	
Yes	840	62.1%	674	60.2%	166	71.6%	
Adjuvant therapy							0.122
Chemotherapy alone	258	19.1%	202	18.1%	56	24.0%	
Chemoradiation	413	30.5%	327	29.2%	86	36.9%	
Radiation alone	169	12.5%	145	13.0%	24	10.3%	
Depth of stromal infiltration							<0.001
<1/2	595	44.0%	572	51.1%	23	9.9%	
≥1/2	757	56.0%	548	48.9%	209	90.1%	
Tumor size (cm)							<0.001
<4 cm	1115	82.5%	983	87.8%	132	56.9%	
≥4 cm	237	17.5%	137	12.2%	100	43.1%	
LVSI							<0.001
No	1058	78.3%	941	84.0%	117	50.4%	
Yes	294	21.7%	179	16.0%	115	49.6%	
Positive vaginal margin							<0.001
No	1316	97.3%	1095	97.8%	221	95.3%	
Yes	36	2.7%	25	2.2%	11	4.7%	
Parametrial extension							<0.001
No	1322	97.8%	1115	99.5%	207	89.2%	
Yes	30	2.2%	5	0.5%	25	10.8%	

### 3.2 Patients survival between cN0 and cN1

**FIGURE 2:**
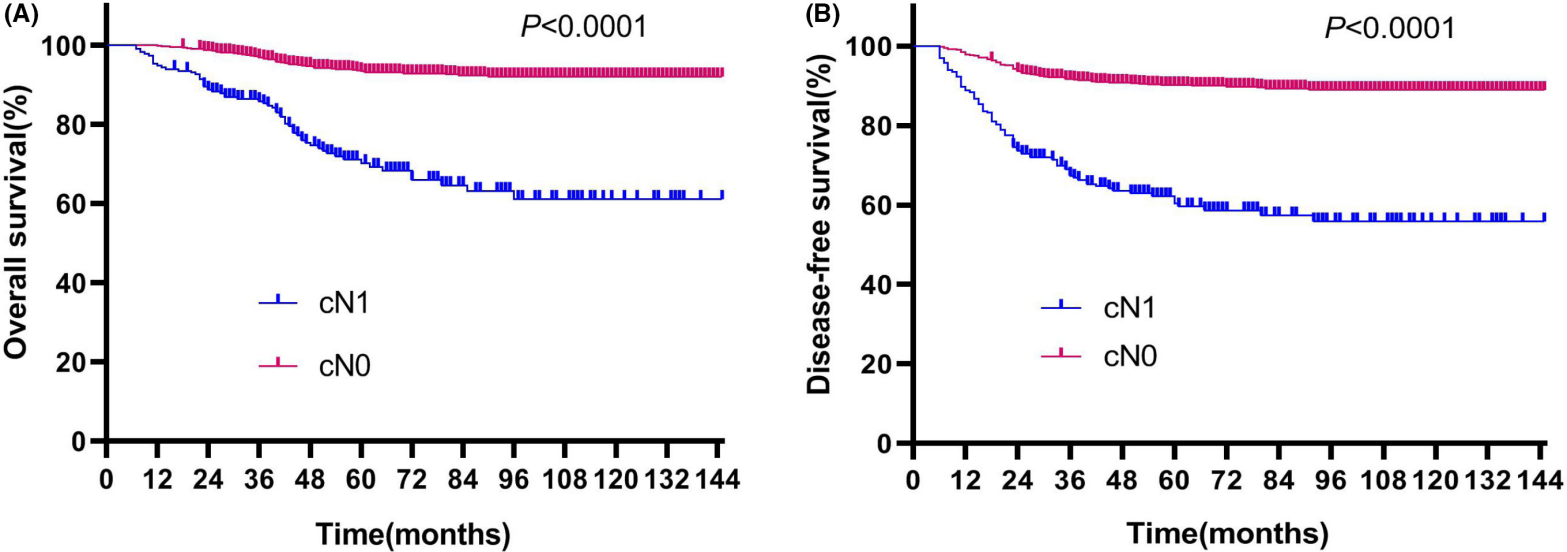
Survival outcome based on lymph node status: (A) Overall survival; (B) Disease‐free survival.

Median follow‐up time for all patients was 82 months (54–114). The mortality rate in cN1 group was higher than that in cN0 group (27.6% vs. 5.9%, *p* = 0.001). Median OS for the cN0 patients was significantly longer (median 61 mo, range 39–91) than the cN1 patients (median 48.5 mo, range 30–72) (*p* < 0.001). The estimated 5‐year and 10‐year OS were 93.5% and 92.1% for the cN0 group, which were significantly longer than cN1 group (71.1% and 61.1%) (*p* < 0.0001), and this significant difference is also reflected in Figure 2A (*p* < 0.0001).

Tumor recurrence was significantly less common in cN0 patients as compared with cN1 patients, (9.7% vs. 38.8%, *p* < 0.001). Patients with cN0 disease had a significantly longer DFS of 60 months (36–90), as compared with 43 months (23–68) in cN1 patients (*p* < 0.001). Patients in cN0 group had the higher 5‐year and 10‐year DFS (90.1% and 88.9%) compared with patients in cN1 group (62.2% and 55.9%). As illustrated in Figure 2B, the DFS rates were significantly different between the two groups (*p* < 0.0001). Moreover, the multivariate analysis also showed lymph‐node status was an independent predictor for recurrence (HR: 3.058; 95%CI 2.193–4.265, *p* < 0.001) (Table S1).

### 3.3 Recurrence and death time between cN0 and cN1

**FIGURE 3:**
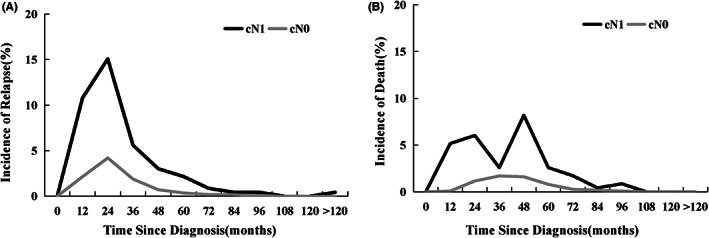
Incidence of (A) recurrence and (B) death by different lymph node status of cervical cancer.

The cumulative rate of relapse and death was also investigated, and a significant difference in their occurrence was observed between the different lymph‐node status (Figure 3A). The incidence of relapse in cN1 group was relatively high in the first year after operation (1‐year relapse rate, 10.78%), and continued to rise to the peak in the second year, then began to decline year by year, and the cumulative rate of relapse reached 36.65% by the fifth year of follow‐up, patients had a significantly reduced risk of relapse 6 years after surgery. However, in cN0 group, patients relapsed slowly in the initial 2 years with a 1‐year relapse rate of 2.14%, a peak in the second year, and decreased year by year from then on, the cumulative rate of relapse reached to 9.29% by the fifth year of follow‐up, and patients had a significantly reduced risk of relapse 4 years after surgery.

The incidence of death during the follow‐up was also different between the two groups (Figure 3B). The cN1 group demonstrated a significant increase in the incidence of death, which peaked in the first 4 years while remained relatively stable after 5 years. Patients of cN0 group demonstrated a smooth and steady increase in death incidence, which remained constant during the 10 years after diagnosis.

### 3.4 Recurrence site between cN0 and cN1

**FIGURE 4:**
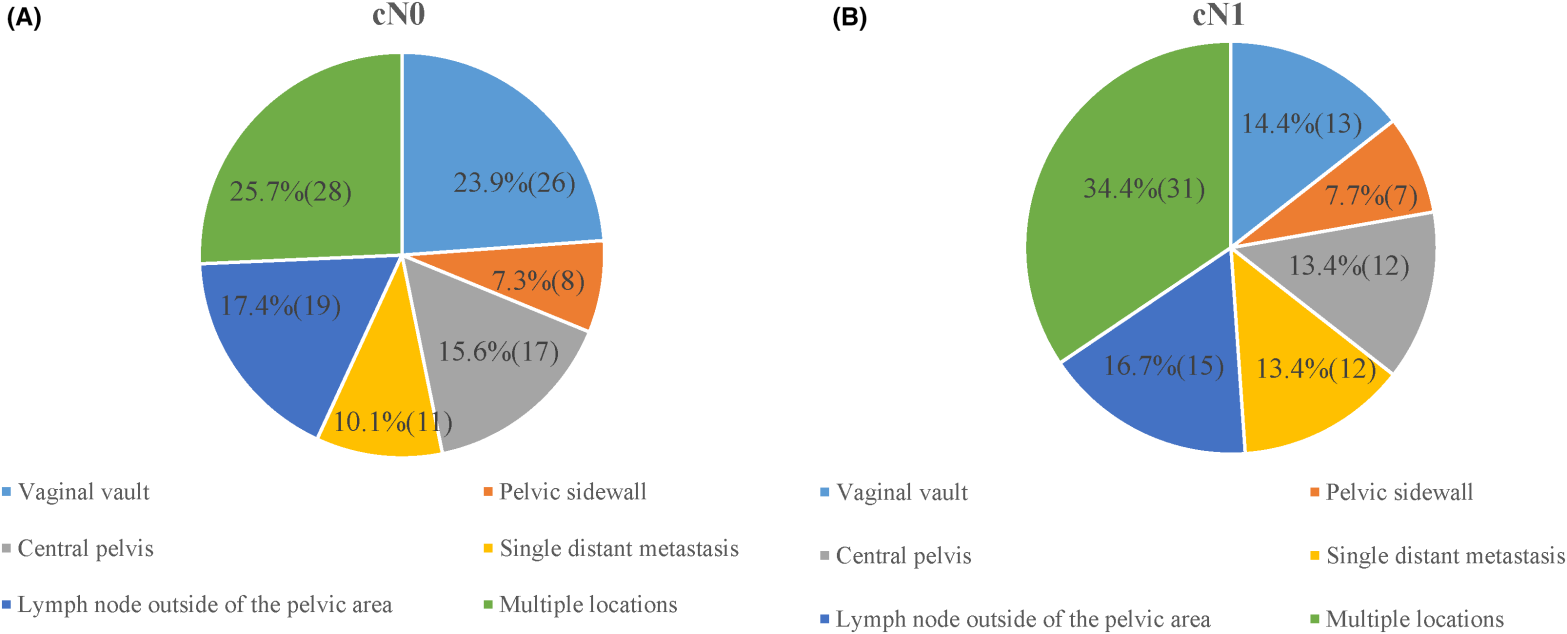
Distribution of metastatic sites by different lymph node status of cervical cancer: (A) cN0 group; (B) cN1 group.

The site of recurrence was analyzed in patients with recurrent diseases in the two groups. We firstly used six classification methods, as shown in Table 2 and Figure 4, the recurrent sites tended to be approximately the same between the two groups (*p* = 0.528). Notably, although the most common site of recurrence was multiple locations (25.7% of cN0 and 34.4% of cN1), in cN0 patients, the frequency of vaginal vault metastasis was 23.9%, which was slightly lower than multiple locations and significantly higher than cN1 group (14.4%), while in the cN1 group, the frequency of vaginal vault metastasis ranked as the third metastatic place, less than that of the lymph node outside of the pelvic area (16.7%). However, in cN0 group, the third metastatic place was the lymph node outside of the pelvic area (17.4%), followed by central pelvis (15.6%), single distant metastasis (10.1%), and pelvic sidewall (7.3%). In cN1 group, there was a slight difference in the 4th and 6th rank of metastatic risk, the fourth is the metastasis of central pelvis and single distant metastasis, which has the same incidence rate (13.4%), the lowest risk place of metastasis was pelvic sidewall(7.7%). Then we use three categories for analysis, as shown in Table 2, recurrence within, beyond, both within and beyond the pelvic cavity were further analyzed. Our research discovered that patterns of recurrence were similar between cN0 and cN1 patients. To be specific, only within pelvic cavity recurrence was the most common first site of disease progression (cN0 44.0% and cN1 38.9%, *p* = 0.646). Only beyond pelvic cavity recurrence occurred in 27.5% of cN0 and 33.3% of cN1 patients. Both within and beyond pelvic cavity recurrence occurred in 28.4% of cN0 and 27.8% of cN1 patients. Furthermore, specific sites of distant recurrence were also similar, with 31.0% of distant metastases to lungs, 20.8% to multiple sites and 13.8% to livers, kidneys and ureter in cN0 patients, whereas of cN1 patients metastasized 25.8% to lungs, 22.7% to multiple sites and 12.9% to the liver, kidneys, and ureter. (Table S2).

**TABLE 2:** Outcome data and univariate analysis of 1352 patients with cervical cancer

Variables	Total *n* = 1352		cN0 = 1120(82.8%)		cN1/*n* = 232(17.2%)		Univariate
	Median/*n*	IQR(%)	Median/*n*	IQR(%)	Median/*n*	IQR(%)	*p‐*value
Disease‐free survival	56	35–88	60	36–90	43	23–68	<0.001
Overall survival	57	38–88	61	39–91	48.5	30–72	<0.001
Recurrence							<0.001
No	1153	85.3%	1011	90.3%	142	61.2%	
Yes	199	14.7%	109	9.7%	90	38.8%	
Recurrent sites							0.528
Vaginal vault	39	19.7%	26	23.9%	13	14.4%	
Pelvic sidewall	15	7.5%	8	7.3%	7	7.7%	
Central pelvis	29	14.6%	17	15.6%	12	13.4%	
Single distant metastasis	23	11.5%	11	10.1%	12	13.4%	
Lymph node outside of the pelvic area	34	17.1%	19	17.4%	15	16.7%	
Multiple locations	59	29.6%	28	25.7%	31	34.4%	
Number of recurrent sites							0.974
Solitary	71	35.7%	39	35.8%	32	35.6%	
Multiple	128	64.3%	70	64.2%	58	64.4%	
Recurrent pattern							0.646
Within pelvic cavity	83	41.7%	48	44.0%	35	38.9%	
Beyond pelvic cavity	60	30.2%	30	27.5%	30	33.3%	
Both within and beyond pelvic cavity	56	28.1%	31	28.4%	25	27.8%	
Death							<0.001
No	1222	90.4%	1054	94.1%	168	72.4%	
Yes	130	9.6%	66	5.9%	64	27.6%	

## 4 DISCUSSION

In recent years, the concept of precision medicine has been highly promoted, which emphasizes the clinical relevance of defining each patient's risk to personalize treatment and follow‐up. Nodal involvement has been found to negatively affect the prognosis of cervical cancer. Although lymphadenectomy allows histological assessment of lymph node status to be obtained, it contributes to a variety of complications. Based on the theory of precision medicine, many studies have explored personalized management of lymph nodes in the treatment of cervical cancer, such as precise definition of the risk of lymph node metastasis for early‐stage cervical cancer patients in order to avoid unnecessary lymphadenectomy^15^ and exploration of minimally invasive surgery for localized lymph node recurrence in cervical cancer.^16^ However, no studies have been conducted about the pattern and timing of recurrence associated with lymph node status so as to provide personalized follow‐up of postoperative patients with cervical cancer. This is the first report to explore the recurrence patterns in cN0 versus cN1 cervical cancer patients. The sites of recurrence showed strikingly similar between cN0 and cN1 groups. Of the patients involved, 27.5%–33.3% presenting distant metastasis, 38.9%–44.0% local recurrence, and 27.8%–28.4% with both within and beyond pelvic cavity (not statistically significant). However, the length of time to recurrence and overall survival showed the difference between cN0 and cN1 patients. Accordingly, the recurrence patterns between cN0 and cN1 showed certain differences, and our research intends to provide clinical evidence to explore the necessity and feasibility of accurate follow‐up based on lymph node status.

It has been well documented that patients with lymph node metastasis showed much higher recurrent risk as compared with patients with lymph nodes negative.^17^, ^18^

Along similar lines, our current study reported lymph metastasis was an independent predictor of recurrence by using univariate and multivariate analysis. We then explored the reasons for the high risk of recurrence in patients with lymph node metastasis and found that cN1 patients were more likely to have later FIGO stages, worse histological grade, deeper stromal infiltration, positive vaginal margin, parametrial extension, LVSI, and larger tumor size, which were consistent with previous reports.^19^ Thus, patients with lymph node metastasis exhibit more aggressive and poorer differentiation. Then, the question is, are there any differences in the recurrence pattern, recurrence time, and location between cN0 and cN1 groups?

Interestingly, sites of recurrence were similar, except for the risk of vaginal vault metastasis. According to the six categories, both in cN0 and cN1 groups, the most common recurrence site in our study was multiple locations, followed by lymph node outside of the pelvic area in cN1 group, while in the cN0 group, the risk of vaginal vault metastasis was only slightly lower than that of multiple locations, and was significantly higher than the incidence in the cN1 group. In the three categories, the most common site of recurrence in both groups was only within pelvic cavity, followed by only beyond pelvic cavity and both within and beyond pelvic cavity recurrence. This conclusion is in accordance with the distribution trend of recurrent sites reported in literature.^20^ In summary, whether there was lymph node metastasis or not, the distribution of recurrence sites was basically the same, and the specific inspection items performed during follow‐up should be basically the same whether the lymph node is positive or not, while unexpectedly the cN0 group had a higher risk of metastasis in vaginal vault than the cN1 group. One potential reason may be that most lympho‐positive patients received postoperative radiotherapy, and pelvic radiotherapy may reduce the risk of recurrence of the vaginal vault. Accordingly, for the follow‐up of patients with negative lymph nodes, special attention should be paid to the examination of the vagina vault in order to avoid missing recurrence cases.

Although there was no significant difference in the site of recurrence, the time to recurrence and overall survival showed significantly longer in the cN0 group compared with the cN1 group. This is consistent with the findings of Gry Assam Taarnhøj et al.^7^ In our study, the majority of relapse cases occurred within 2–3 years after the last primary treatment, and almost no recurrence occurred after 5–6 years, which is consistent with previous reports.^13^, ^21^, ^22^ NCCN guidelines recommend the following follow‐up strategies for patients with cervical cancer: about the interval of follow‐up, every 3–6 months for 2 years, every 6–12 months for 3–5 years, then annually based on the patient's risk of disease recurrence. Although close follow‐up facilitated early detection of potential recurrence, it is important to determine the optimal follow‐up interval from a health economics perspective. According to our data, follow‐up of patients with cervical cancer may be individualized based on their risk of recurrence. Patients with stage I‐IIA cervical cancer should have close surveillance during the first 2 years after radical surgery. According to our results, in the first year after surgery, patients with positive lymph nodes, need a more frequent follow‐up, such as every 3 months, while patients with negative lymph nodes may be considered for follow‐up every 6 months. In the second year after surgery, both cN0 and cN1 cervical cancer patients showed a high risk of recurrence, so follow‐up conducted every 3–6 months is highly recommended. In the third year after surgery, patients with cN0, need follow‐up every 6 months, while patients with cN1 may need a more frequent follow‐up interval, for example, follow‐up every 3–4 months. In the 4–5 years after surgery, an annual follow‐up would be sufficient for patients with cN0, however, patients with cN1 need follow‐up every 6 months. Moreover, while patients with cN0 can be considered cured if there is no recurrence 5 years after the surgeon, patients with cN1 can be considered cured if there is no recurrence after 6 years of follow‐up. Our data may build the basis for a prospective surveillance trial to determine the optimal strategy to follow‐up patients with different risk of recurrence.

There are potential limitations of our study however. First, this is a retrospective study that may miss confounding factors. Our study was the result of a long‐term follow‐up at a single center, routine follow‐up visits may delay the detection of recurrence because some patients may not present symptoms until their next routine appointment. In the present study, we confirmed recurrence by conducting a pathological review and/or secondary imaging evaluations, thus not all recurrence cases were pathologically confirmed. And the results of the current study warrant further investigation in prospective studies to validate our stratification method. Any effect on survival outcomes would necessarily require prospective evaluation.

## 5 CONCLUSION

Our study showed that patients with cN1 showed a higher risk of recurrence compared with cN0 cervical cancer patients. The time to recurrence was significantly longer in cN0 patients compared with cN1, however, there was no significant difference in the distribution of recurrence sites, except that cN0 patients were more likely to have vaginal fornix metastasis compared with cN1 patients. Our results may help to obtain optimal follow‐up strategies according to their pattern of recurrence based on lymph node status. More frequent follow‐up intervals are recommended for lymph node‐positive patients than for node‐negative patients, except for the second year postoperatively when the frequency of follow‐up is the same for lymph node‐positive and node‐negative patients. For patients with negative lymph nodes, special attention should be paid to the examination of vaginal fornix during follow‐up.

## AUTHOR CONTRIBUTIONS

Mei Ji performed data analysis, reviewed the literature, and drafted the article. Yuan Liu and Yan Hu collected clinical data. Haiyan Zhu designed the study and finalized the paper. Jing Sun also participated in the design of the study and provided suggestions to improve it. All authors contributed to the article and approved the submitted version.

## FUNDING INFORMATION

The research was supported by grants from Specialized Clinical Research in Health Industry of Shanghai Municipal Health Commission (202040129), the Science and Technology Commission of Shanghai Municipality (19411960300), the Shanghai Municipal Health Commission (2019SY002), and the Shanghai Hospital Development Center (SHDC12019113).

## CONFLICT OF INTEREST

To the best of our knowledge, the named authors have no conflict of interest, financial or otherwise. We declare that: (1) no support, financial or otherwise, has been received from any organization that may have an interest in the submitted work; and (2) there are no other relationships or activities that could appear to have influenced the submitted work.

## ETHICS STATEMENT

This study was approved by the Ethics Committee of the First Affiliated Hospital of Wenzhou Medical University and informed consent was signed by the patients before surgery.

## Supporting information


**Table S1.** Multivariate survival analysis of 1352 patients with cervical cancer

**Table S2.** Distant recurrence distribution

## Data Availability

All data included in this study are available upon request by contact with the corresponding author.
